# Reporting the Social Value Generated by European Universities for Stakeholders: Applicability of the Global Reporting Initiative Model

**DOI:** 10.3389/fpsyg.2021.787385

**Published:** 2021-12-13

**Authors:** Jorge Gutiérrez-Goiria, Iratxe Amiano-Bonatxea, Antonio Sianes, María José Vázquez-De Francisco

**Affiliations:** ^1^Hegoa Institute, University of the Basque Country (UPV/EHU), Bilbao, Spain; ^2^Research Institute on Policies for Social Transformation, Universidad Loyola Andalucía, Córdoba, Spain; ^3^Fundación ETEA Development Institute, Universidad Loyola Andalucía, Córdoba, Spain

**Keywords:** GRI standards, sustainability reports, social value, stakeholders, higher education institutions, societal challenges, 2030 Agenda, comparative case study

## Abstract

Universities are increasingly being asked to contribute to addressing the significant local and global challenges, such as those identified in the 2030 Agenda. Set in this framework, universities need to account for the social value they generate through their activities, particularly from the perspective of their contribution to different stakeholders. This approach requires, first of all, that the main stakeholders are identified. Relationship and dialogue mechanisms then need to be established which can help guide universities to choose activities which can better meet the needs of their stakeholders. The current paper analyses the potential of integrated reports, and triple bottom line reports, as an instrument for reporting on aspects that go beyond the financial sphere, including economic, social and environmental aspects. Specifically, the paper focuses on studying the viability of the Global Reporting Initiative (GRI) for reporting the value that European universities generate for their stakeholders. The results show, firstly, that the universities in the sample do not sufficiently address these questions in their reports. Internal stakeholders are prominent in their reports, with the interaction between them and the universities being generally unidirectional. References to value generated are limited, and usually refer to the economic value. However, some examples of good practices are identified that could be used to improve standards of reporting, especially in universities committed to integrated reporting initiatives, in order to better reflect the social value.

## Introduction

Challenges, such as the increase in social inequalities or the worsening of the ongoing environmental crisis, are prompting society to look to the social role of universities to address these problems. Their involvement is now considered essential in helping to find a solution to the significant local and global issues, such as those identified in the 2030 Agenda (Utama et al., 2018; Sianes, 2021). The academic literature is beginning to identify the components of a new way of viewing a university’s activity in which the priority lies in generating value for the stakeholders involved (Tetteh et al., 2021). These debates are putting forward different proposals which advocate for the potential of universities to be at the service of society.

In this context, disclosure of the broader impact that universities have through their role is fundamental. This has given rise to academic reflections as well as practical advances in suggesting new models for Higher Education Institutions (HEIs) to use to be able to collate and communicate financial and non-financial information.

At present, the financial statements completed by HEIs do not allow for the value created by these institutions to be accounted for (Adams, 2018), yet the latest trends promote the elaboration of ‘integrated reports’ or ‘triple bottom line reports’, capable of reporting economic and social information in the same document. It is hoped that, by using these reports, universities can move from a financial accountability approach to an approach that incorporates recognition of the value they generate for their stakeholders, demonstrating how this value is created and the components involved.

To achieve this, report models are proposed based on processes in which information gathered through an ongoing dialogue with stakeholders is collated and recorded in a memoire or public report, and given as much value in the report as traditional accounting. One widely used report model, which goes beyond financial aspects, is that proposed by the Global Reporting Initiative (GRI).

This study begins with a review of the GRI as a model and framework for integrated reporting in a broad sense. It then goes on to analyse the reports prepared by different European universities, which are using the most recent version of GRI Standards, by posing the following questions:

- Which stakeholders do universities take into account in their reports? How do they evaluate the relationship (engagement) with stakeholders? What procedures are in place for this?
- How do they approach the generation of value in their reports? What types of value do they consider? Do they relate the generation of value to the stakeholders and their needs?
- Is the GRI model adequate as an instrument for reporting stakeholder participation and involvement, as well as for conveying to stakeholders the social value generated by the university’s endeavours?

With these questions as a guide, Section “Generating and reporting value for stakeholders in Higher Education Institutions” introduces the theoretical framework, based on three aspects: debates on the value generated by universities; consideration of the stakeholders and their relationship with the institution as key aspects for guiding the management of the universities and the generation of value; and the existing reporting models, focusing on those that go beyond economic values, such as the GRI methodology which this current study analyses. Section “Methodology” details the methodology and the sample analysed, while Section “Results” shows the results obtained for the universities studied. After section “Discussion,” the article ends with conclusions.

## Generating and Reporting Value For Stakeholders in Higher Education Institutions

### Generating Value in Higher Education Institutions

In a framework of social and economic change accelerated by globalisation, the social problems and challenges that begin at a local level quickly reach a global dimension (Stiglitz and Greenwald, 2014). In the context of the social model known as the ‘knowledge society’ (World Bank, 2002), society appeals to the university, as an institution that generates knowledge, to propose alternatives and respond to new global problems and challenges (Altbach, 2008; UNESCO, 2009; Boni and Gasper, 2012; Cardona et al., 2016; Grau et al., 2017).

The university, often through its social responsibility policies, sees its mission as being at the service of society. Since the 1990s, academics and researchers have supported the role of the university as a generator not only of economic value but also of social value (Stokes and Coomes, 1998). Through diverse actions related to teaching, research and knowledge transfer, the university generates value and affirms its social commitment (Dima, 2015; Wallace and Resch, 2017) and its commitment to human development (Oketch et al., 2014; Boni and Walker, 2016).

Some authors identify value generation in line with the companies where graduates will be employed (Kucharcikova et al., 2019). Other authors highlight the value that is generated through the transfer of knowledge, or the co-creation of knowledge with other agents, which generates an impact on the economic and social environment (Meissner and Carayannis, 2017; Di Nauta et al., 2018; Djikhy and Moustaghfir, 2019). More recently, certain authors analyse the value contributed by the university as a co-transforming agent and catalyst of innovation in its local environment (Ponsiglione et al., 2018). The public procurement process of universities, as a way of generating value in the economic environment, is also analysed from a critical perspective by the academic literature (Torres et al., 2010; Hofacker et al., 2012; Valero and Van Reenen, 2019).

Van den Akker et al. (2017) analyse how the response of universities to social challenges is changing the general view of the impact these institutions can have, and this is influencing their research and teaching. Academic work is framed from this perspective in a dynamic in which the contribution of the different stakeholders is considered and their involvement is crucial in working towards a common goal.

In addition, the way of financing universities has changed dramatically in recent decades, which has led to a rethinking in terms of how they generate value and translate it into sources of income. These new areas of work, closely related to innovation and economic development, also require new types of relationships between the university, social agents and governments (Etzkowitz and Leydesdorff, 2000; Jaegersberg and Ure, 2017; ONeill et al., 2017).

In the current context of commercialisation of university activity that Slaughter and Leslie (1997) described, some authors emphasise that valuation is broader than commercialisation. In the words of Benneworth and Jongbloed (2010, p. 568), the way of measuring the value generated by universities based solely on their economic contributions can often overshadow the broader contributions to society.

However, appropriation of the value generated by the universities is not without criticism. Within the framework of the knowledge economy, this generation of value brings with it, for example, a discussion about the legitimacy with which the value generated in laboratories or universities is distributed (Iscaro, 2014; Veltri and Silvestri, 2015).

In any case, the generation of value by universities only makes sense when it is perceived by their stakeholders. Thus, some authors have made proposals that enable the identification and measurement of the value generated by entities in general (Hartmann and Ibanez, 2006; Mittal et al., 2008; Kocmanová et al., 2016; Evans et al., 2017) and, more specifically, by universities (Sánchez-Fernández et al., 2010; Fagerstrøm and Ghinea, 2013; Lubik et al., 2013; Mindruta, 2013; Booth and Kellogg, 2015; Ayuso et al., 2017).

The methodologies proposed by different authors to identify and/or measure the value that is generated are developed within the framework of the Stakeholder Theory (Friedman and Miles, 2002; Freeman et al., 2010; Garriga, 2014; Tantalo and Priem, 2016).

### Stakeholder Theory in the University Environment

Stakeholder Theory has been used in the literature to justify the incorporation of new management systems into the university environment, especially in the incorporation of improvements in its governance and in the decision-making processes at an institutional level (Saurbier, 2021).

The interpretation by Larrán-Jorge and Andrades-Peña (2015) of this theory is that it incorporates the commitment of universities – through teaching, research and knowledge transfer – to meeting the needs of the different stakeholders from three aspects: economic, social and environmental. Dialogue must be recognised as an enabler of legitimacy and transparency based on symmetrical communication between stakeholders.

The idea of symmetry has caught the attention of a number of studies which analyse the nature of the relationships that universities establish with their stakeholders. Mainardes et al. (2012) study not only the presence but also the relevance of the different stakeholders in the university context, for which they apply the theory of stakeholder identification and salience of Mitchell et al. (1997) to the university setting, adding an additional attribute, influence. The results of their study show that the influence between the university and its stakeholders cannot be measured by four factors alone (one only influences, one is only influenced, one influences and is influenced, one does not influence and is not influenced), but rather the relationships should be evaluated in a much broader sense, taking into account whether these entities and institutions are influenced more than they influence or vice versa.

Along these lines, Benneworth and Jongbloed (2010) show how organisations establish links of a different nature with their stakeholders, paying more attention to certain stakeholders than others, depending on the nature of the link established. However, these links are not formally established, so they are neither static nor the same in all organisations and the context and reality of each organisation will determine the composition and relevance of the link established with each stakeholder (Pantoja et al., 2015).

The influence that the different stakeholders have on university activity largely depends on the objective of their relationship. In this sense, Duque (2009) highlights that the university has given more importance to its links with those interest groups related to the economic system than other interest groups related more to the social system.

By adopting the sustainability approach in their management strategy, universities consider identifying their stakeholders from sectors that may have expectations of value generated based on the role of the university, either in its internal aspect (students, staff contracted) or in its external aspect (suppliers, companies, public administrations, etc.). However, what really represents an innovation in Stakeholder Theory in the university environment is the interaction with other agents of society, beyond those with whom there is a specific functional relationship (Whitmer et al., 2010). Studying the scope of what universities consider to be their stakeholders and the quality of their communication channels is essential if universities want to know what value they generate for these interest groups.

But, as the academic literature shows, in addition to identifying the value generated by universities, this value must also be communicated to society. Numerous academic articles focus attention on this aspect, and this is discussed in the next section.

### From Financial Reporting to Integrated Reporting as a Way of Accounting for the Value Generated

Traditionally, financial accounting has served as an instrument for disclosing relevant information about organisations through the publication of their financial statements. In recent decades, entities have shown themselves to be more committed to major global challenges, and they take into account the impacts of their activities on these issues in their strategic management. Nowadays, in addition to generating profits, organisations are expected to contribute to the creation of global value.

In this context, accounting information systems have had to evolve to respond to a society that demands greater transparency in terms of performance, not only economic but also social and environmental. Social accounting is a relatively new development in the accounting world, first appearing in the business sector in the mid-1950s (Mook, 2014).

In the 1990s, interest in social accounting resurfaced and new models of accountability were proposed that were capable of integrating social accounting in the management and accounting systems of organisations. Current trends indicate that organisations are choosing a new way of communicating their social and environmental policies, prioritising the mitigation of information asymmetry between users (Rodrigues et al., 2021).

Indeed, in a current scenario where the 2030 Agenda and the Sustainable Development Goals (SDG) have become the objectives shared by all types of organisations, the financial statements proposed through integrated social accounting put social and environmental performance alongside financial performance (Mook, 2020).

Integrated information represents a major change in the disclosure of information in organisations, and regulatory bodies are becoming more aware of the complexity of the current business model. The acceptance of non-financial metrics to reflect the creation of value is considered progress towards better communication of results to stakeholders (Burke and Clark, 2016).

Organisations with responsibility for the standardisation of information made public by institutions [such as the American Institute of Certified Public Accountants (AICPA), International Integrated Reporting Council (IIRC), International Accounting Standard Board (IASB) and International Organisation for Standardisation (ISO)] are working on proposals that allow the integration of financial and non-financial information in the same document, with appropriate adaptations for certain sectors (De Vicente-Lama et al., 2021).

Integrated reports make it possible to provide a holistic view of an entity, acknowledging and communicating the total value of an entity to both internal and external users. One of the benefits derived from the preparation of this type of report is that it can be used for interaction with interested parties (Burke and Clark, 2016).

Although the fundamental pillar of the integrated report is the traditional financial report, regulated by accounting standards and required by legal regulations, the main initiatives that promote the preparation of integrated reports are based on developments that complement regulated financial information with other types of information that give insight into the social and environmental impact of organisations. Thus, several authors present integrated reporting as a further step in the sustainability reporting process (Stubbs and Higgins, 2014; Brusca et al., 2018).

Ogata et al. (2018) find a co-dependent relationship between sustainability reporting and integrated reporting, which means that sustainability reports, such as those based on the GRI principles, can be useful in disseminating the activities of entities from an ESG (Environmental, Social and Governance) perspective. In this sense, the GRI standards, widely recognised internationally, are designed for the presentation of sustainability information that can be included in integrated reports. Currently, the GRI Reporting Framework has a main role in the process of preparing these types of reports (Michalczuk and Konarzewska, 2018).

The Higher Education sector is also keen for society to recognise the efforts made and the value generated, and therefore places great importance on the preparation of reports typical of social accounting. Since the financial statements currently in use are not capable of transmitting the value created (Adams, 2018), universities are addressing this by actively producing sustainability reports or documents. These reports sometimes follow their own models, and on other occasions they take advantage of those models that have already been standardised, the GRI being the one most widely implemented (Alonso-Almeida et al., 2015; Amiano et al., 2021).

The GRI, created in 1997 as a joint project between the United Nations Environment Programme (UNEP) and the Coalition for Environmentally Responsible Economies (CERES), incorporates a set of standards with social and environmental content that, in the case of universities, allows them to gain more visibility, improve their reputation in certain areas, and attract new financing (Lozano, 2006; Larrán and López-Hernández, 2010; Rojas Donada and Bernardo Vilamitjana, 2016).

The objective of GRI is to create a global standard for sustainability reports so that they are rigorous and comparable, with a style similar to that of financial reports. Its scope, initially very focused on sustainability, has been extended to social, economic and governance objectives.

In practice, few universities publish sustainability reports. Obstacles to this include the lack of adaptation of reporting models to this sector (Alonso-Almeida et al., 2015; Ceulemans et al., 2015), which requires a contextualised approach. Along the same lines, Amiano et al. (2021) show the difficulty of the GRI methodology for reporting on issues related to the central objectives of universities.

One of the basic principles in the GRI methodology calls for an organisation to identify and explain how it responds to the expectations and interests of its stakeholders. This includes those who cannot make their views heard and whose concerns need to be channelled through their representatives (e.g., through NGOs), and also those with whom the organisation cannot maintain an open and ongoing dialogue. Emphasis is placed on identifying the legitimately established needs of society.

Among the information to be documented, GRI asks for an explanation of how stakeholder participation has influenced not only the content of the report but also the activities, products or services provided by the organisation (Global Sustainability Standards Board, 2016a).

The GRI methodology, as discussed in the empirical contrast, incorporates specific disclosures that can be used to report the creation and distribution of the economic value generated in organisations. These variables, typical of social accounting, have been developed by the GRI to reflect the importance it places on organisations explaining how they have generated value for stakeholders (Global Sustainability Standards Board, 2016b).

## Methodology

The state of the art has shown that, at present, there are still numerous ongoing debates on how universities should integrate their stakeholders and generate value for them. From these debates, the following research questions are derived to guide this empirical study: which stakeholders do the HEIs take into account in their reports and how do they interact (engage) with them? How do they address the generation of value in their reports and how do they relate it to stakeholders and their demands? The review also highlights that universities have begun to use different integrated reporting models, introducing them into their management processes, although the model most used, which emerged in recent years and which continues to gain strength, is the GRI Standards model. This gives rise to the last question that guides this study: does the GRI model prove itself to be an adequate instrument for reporting on these aspects of university management?

To address these research questions, the reports published by universities adopting the latest GRI version available, GRI Standards, are used. Although any specific selection of reports may limit the possibility of generalisation of the results, for the purpose of this study, only the reports published by universities located in Europe are chosen for analysis. There are several reasons for this choice. They all share a common university regulation, the European Higher Education Area, which means biases of a normative nature are avoided, and all of them are based on a university tradition that is sufficiently similar as to make comparisons possible, but at the same time with their own cultural nuances that can enrich the results. The number of reports available (10) allows a comparative analysis large enough to extract qualitative knowledge of depth and value.

Analysis of the sustainability reports focuses on the most recent GRI report published by each institution, at the time of writing (June 2021). As can be seen in Table 1, the 10 reports analysed belong to HEIs from only four European countries. In three cases (IUNR, ETSII-UPM and ESADE), these are not global reports but rather sustainability reports published by these three faculties or institutes independently of the university to which they belong. All the institutions analysed are public universities except for one private entity (ESADE). Only one of the selected HEIs uses the ‘comprehensive’ GRI report format (University of Cádiz). The remaining reports use the shorter model (core report).

**Table 1:** Description of the sample.

University	Country	Reported period	Date of publication	GRI report	Ownership
Università di Firenze	Italy	2018	2019	Core	Public
Università degli Studi di Torino	Italy	2018/19	2018	Core	Public
Fundación ESADE (Universidad Ramón Llull)	Spain	2018/19	2020	Core	Private
Universidad de Cantabria	Spain	2015/16, 2016/17	2018	Core	Public
Universidad de Cádiz	Spain	2018/19	2020	Comprehensive	Public
Universidad Politécnica de Madrid (ETSII-UPM)	Spain	2016/17	2019	Core	Public
ETH Zürich	Switzerland	2017/18	2019	Core	Public
Universität Zürich	Switzerland	2018	2019	Core	Public
IUNR Institut für Umwelt und Natürliche Ressourcen (IUNR-ZHAW)	Switzerland	2017/2018	2019	Core	Public
Manchester Metropolitan University	UK	2017/18	2019	Core	Public

*Source: authors´ own elaboration*.

The method used in this study is the comparative case study (Bartlett and Vavrus, 2016), which is ideal for this type of comparative analysis. By comparing cases, potential patterns in the use of GRI reports can be detected, identifying similarities and differences while facilitating the extraction of specific results from each institution that can serve as good practices in the sector. The use of the comparative case study covers, therefore, both academic learning based on the systematisation of empirical evidence and the extraction of applications for industry.

The first part of the analysis focuses on the role of stakeholders in universities and their ability to influence them. With the aim of making a critical judgement of it, this paper proposes an adaptation of the participation ladders developed by authors, such as Arnstein (1969). To this goal, and inspired by similar proposals, such as those by Moratis and Brandt (2017) and Ferrero-Ferrero et al. (2018), the instruments through which universities interact with their stakeholders have been classified into two types: one-way instruments, which encompass all those that the university uses to inform and communicate their activities to stakeholders and two-way instruments, which encompass all those in which stakeholders have the capacity to respond and interact. To better analyse this latter type of bidirectional relationship, three categories are created as: consultation instruments through which the university consults the opinion of the stakeholders before making a decision; dialogue instruments, in which the university generates a space for exchanging opinions with stakeholders before making a decision; and co-decision instruments, in which the university and the stakeholders co-decide or co-execute the agreement reached after their dialogue.

The second part of the analysis evaluates how universities integrate the generation of value for such stakeholders in their reports. To this goal, a differentiation between economic value and social value is followed.

To identify how universities report these aspects of their management, the following process is implemented. Firstly, disclosures directly related to each topic (stakeholders and generation of value) are examined. Afterwards, to complement this analysis with an inductive approach, selected keywords (“stakeholders” and “value”) are defined and searched across the reports. Then, information on the relationship with stakeholders and the generation of value for them is located, taking note of the disclosures where universities report these issues. Finally, these additional disclosures where universities account for these topics are re-examined in all universities. The combination of this deductive and inductive approach contributes to answer the third research question, about the potential of GRI Standards as an adequate guide for universities’ reporting.

The results from applying this procedure to the aforementioned GRI reports are presented in the next section, following the order described above.

## Results

### Acknowledgement of Stakeholders in the University Reports

#### Identifying Stakeholders

The principles of the GRI methodology give a central role to the relationship that organisations establish with their stakeholders. Of the 10 universities in the sample studied, only 3 of them (Torino, Cádiz and IUNR-ZHAW) specifically define what they consider to be stakeholders in their activity. Their definitions agree in considering a stakeholder as being exposed to the factor of influence, either to the extent that they influence the activity of the university or that they are influenced by it.

**Figure 1:**
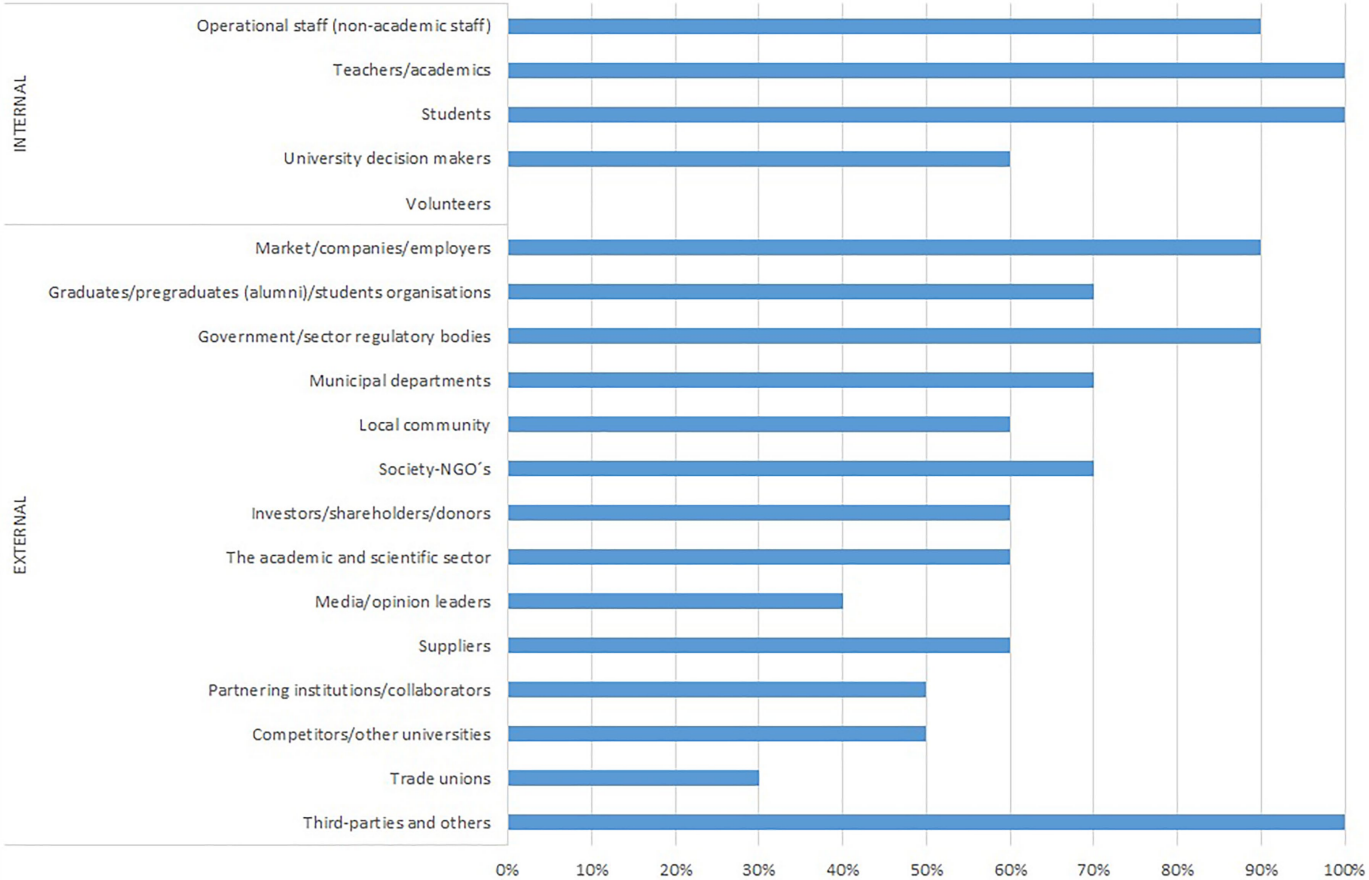
Universities reporting identified stakeholders by category (102–40 disclosure; %).

The number of stakeholders identified by the different reports varies from seven identified by the IUNR-ZHAW to more than 40 identified by the Università di Torino. Although the level of detail with which each of these stakeholders is described in each report varies significantly, it is possible to make an initial grouping of the type of stakeholders identified. Based on the Ferrero-Ferrero et al. (2018) classification, Figure 1 shows the stakeholders that are explicitly identified in the reports that make up the sample.

Figure 1 shows that internal stakeholders are significantly more represented, although two results within this group show that only 60% of the universities add university decision-makers to the usual students, academics and operational staff and no university mentions its volunteer collaborators. With regard to external stakeholders, a wide variety is seen, with very different types of relationships and roles, although there is clearly a greater presence of market institutions and companies (90%). Likewise, 90% of the reports feature the stakeholder Government/Sector regulatory bodies.

These results suggest that the ability to influence how a university generates value depends on the way in which these relationships are established with its stakeholders, an aspect that is explored in the next section.

#### Channels of Relationships and Engagement With Stakeholders

In line with that established in the GRI methodology, another aspect which should be included in the report is the way in which universities interact with stakeholders, in order to identify the objectives (material topics) and the way these activities are developed. However, the detail with which this information is presented differs substantially between universities.

The University of Cantabria, the Polytechnic University of Madrid (ETSII-UPM) and the University of Cádiz detail the channels used for each stakeholder, while ETH Zürich, Manchester Metropolitan University and the Universität Zürich go further, detailing the topics identified for each of them. Other universities, however, provide more vague and generic information regarding the instruments used for dialogue with stakeholders.

**Figure 2:**
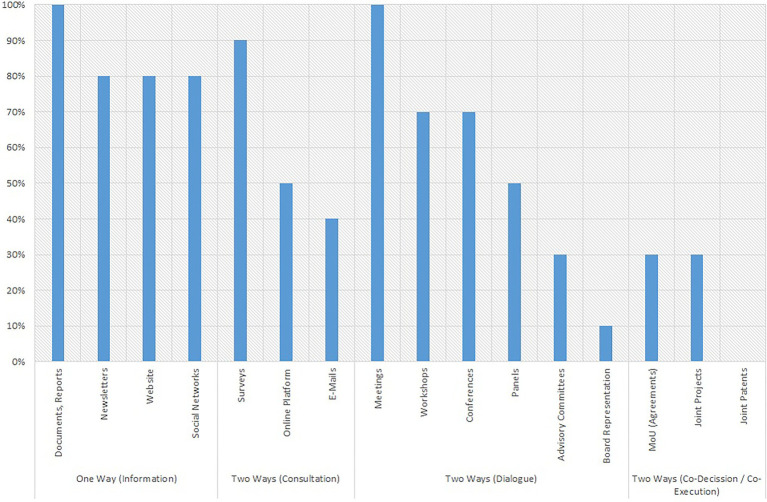
Universities reporting communication with stakeholders by communication channel (%).

Figure 2 shows the different communication channels used by the universities in the four categories mentioned and shows what percentage of the sample has made use of each of these coordination instruments in their relationship with their stakeholders, following the categorisation introduced in the methodology section.

Figure 2 shows a decreasing slope, which suggests that the link between universities and their stakeholders is more informative than participatory. In fact, this categorisation clearly shows that all universities, through one instrument or another, use unidirectional communication channels, which allow them to provide information to their stakeholders. The instruments for consultation and dialogue with stakeholders are also quite popular, especially through meetings, conferences or workshops, which is a good way of obtaining feedback on the specific issues that universities raise with their stakeholders. Up to 90% of the reports refer to having consulted with their stakeholders in order to obtain information on specific topics of interest for the universities. However, this process is not enough to assess the relationship between both actors as bidirectional, as pointed out by Pantoja et al. (2015).

In fact, the data show that only 30% of the universities in the sample acknowledge in their reports the presence of more stable channels that allow their stakeholders to in some way influence university activity, as indicated by Mitchell et al. (1997). When this occurs, it is generally a case of partnership in the implementation of specific projects, since in no case are stable partnerships reported.

### Aspects Related to the Reporting of Value Generated

The principles of the GRI methodology suggest that, in order for these to enable an integrated reporting exercise, organisations should record in their reports the economic and social value generated by their activity. Although *a priori* one would expect universities to make use of their activity reports to declare their contribution to the socio-economic development of the territory, it is clear from the review that reporting of this information is scarce.

In fact, even the information most readily available, that of the annual accounts, is not recorded by 30% of the universities in their reports, and only 60% of them detail the sources of financing that have allowed them to carry out their activity.

There are very few references to the generation of economic value in integrated reporting exercises, and the presence of the other essential element – that of the generation of social value – is even more scarce. Using a detailed keyword search, some references to the social value generated could be found, as summarised in Table 2. This shows the importance given, or not, to this point in the reports.

**Table 2:** References to value for stakeholders and social value in the reports.

Università di Firenze	The question of value (economic and social) is present in different places: on 4 occasions, the report mentions social value generically. It presents a definition of the added value to the stakeholders (valore aggiunto), defining it as an economic-social value that represents the wealth produced and distributed by the university, taking into account the remuneration of all the participating actors. It includes a reclassification of the economic value to calculate the contribution of value to the different stakeholders, including percentages for each case.
Università degli Studi di Torino	The question of value (economic and social) is very present in different places: on up to 8 occasions there is talk of the direct and indirect value that the work of the university adds or transfers to society or its stakeholders. The value generated in relation to the subsidies received is calculated, indicating that the University generated 2.93 euros for the territory with its multiplier effect. A calculation of the direct economic value generated for different stakeholder groups is included (p. 120).
Fundación ESADE (Universidad Ramón Llull)	An isolated mention regarding the alumni mission to generate value for its members and society, without quantification (p. 177).
Universidad de Cantabria	There is no reference to social value. References to the generation of economic value include a breakdown of the direct economic value generated and distributed by concepts (p. 18).
Universidad de Cádiz	It includes specific references to the value chain and aspects, such as those related to purchases (p. 27). It collects information on a Chair of entrepreneurs to co-create economic and social value. It includes an analysis of purchases in proximity and payroll, which partly reflect the economic value contributed in these cases (p. 31–32).
Universidad Politécnica de Madrid (ETSII-UPM)	There are references to the ETSII-UPM value chain and the contribution they make to society from different missions, but it is not quantified (p.84). Some generic reference to the need for value to be transferred to society (p. 87). Some generic reference to added value in strategic alliances.
ETH Zürich	An isolated mention of the value that is generated by saving resources and their reuse (p. 69).
Universität Zürich	There are no direct references to social value, but reference is made to a study on added value by UZH, which indicates that each franc invested produces around 5 (p. 77).
IUNR Institut für Umwelt und Natürliche Ressourcen (IUNR-ZHAW)	No references to social value found
Manchester Metropolitan University	No references to social value found

*Source: authors´ own elaboration*.

As Table 2 shows, reporting on the generation of value beyond the economic sphere, or its distribution among stakeholders, is not a relevant issue for most of the universities in the sample. The mention and identification of social value, or the valuation of aspects related to university activity, are scarce in these extensive reports.

In four universities (ETH Zürich, ESADE, IUNR-ZHAW and Manchester), there is hardly any mention of this aspect. In the case of ETSII-UPM, there are some references to the value chain, and the contribution made to society from different missions. On very few occasions an amount of added value is calculated with respect to the funds received, which is understood as a multiplier (around three in Firenze and five in Zürich) that could represent the socio-economic value generated or returned to society. And only in some cases, already mentioned, the economic information is presented in such a way that it is possible to identify the value generated for some specific stakeholders (e.g., salaries and taxes).

It should be noted that the Italian universities (Firenze and Torino) go one step further by presenting a breakdown of the value generated for stakeholder groups. Despite being fundamentally economic, it reflects an interest in bringing together the issues of value and stakeholders, in what could be an example for other cases.

### Assessment of the Capacity of the GRI Model to Capture the Social Value Generated and Its Transfer to Stakeholders

The previous sections demonstrate that preparation of reports following the GRI methodology has allowed the universities in this analysis to account for the value they generate for society, and show how this value is transferred to their closest stakeholders. This section explores whether the instrument proposed by GRI to achieve integrated reporting, the disclosures, acts as an appropriate guide for universities to communicate these aspects.

First, the GRI contents used by the universities in their triple bottom line reports are shown. Disclosures are divided into general (100), economic (200), environmental (300) and social (400) issues, and Table 3 shows the relative use of each family of disclosures in the reports analysed.

**Table 3:** GRI disclosures used in the reports of the sample universities.

Type of standards	Number of disclosures	Maximum possible	Percentage
100 (universal)	414	590	70.17%
200 (economic)	38	170	22.35%
120	320	37.50%
400 (social)	102	400	25.50%
TOTAL	674	1,480	45.54%

*Source: authors´ own elaboration*.

As can be seen in Table 3, economic contents are the least used in absolute and relative terms (altogether only 22% of the possible disclosures are used), followed by social disclosures (with 25% being used). This initial analysis could already explain why the previously analysed elements of information end up so spread out in the report and not always easily identified.

#### Analysis of the Usefulness of Disclosures Related to Stakeholders

The GRI considers it essential that the report collects detailed information on the composition of its stakeholders and how the organisation relates to them. For this reason, GRI 102 (General disclosures) dedicates a whole section, to detailing Stakeholder engagement. This section includes five disclosures (from 102–40 to 102–44) in which the following information is required as: 102–40 List of Stakeholders; 102–41 Collective bargaining agreement; 102–42 Identifying and selecting stakeholders; 102–43 Approach to stakeholder engagement and 102–44 Key topics and concerns raised. It should be noted that these disclosures must be incorporated in all reports, whether the organisation uses the Core option or the Comprehensive option. Therefore, it is no surprise that all the universities in the sample respond to these disclosures, in greater or lesser detail, except for one university report, which does not include the information related to the Collective bargaining agreements.

In addition to these specific disclosures, stakeholders are also explicitly cited in other fundamental disclosures, such as 102–21 (Consulting stakeholders on economic, environmental and social topics), or even in thematic disclosures, such as 413 (Local Communities), in which the projects undertaken that relate to vulnerable groups can be described, with collective rights, and social projects with an impact on the local community and that take into consideration stakeholders in a broader sense, including those with whom there is no direct relationship. However, these disclosures are not widely used, with only 30% of the universities providing information on 102–41, and 40% of the universities in the sample reflecting their impact on the local community through disclosure 413.

#### Analysis of the Usefulness of Disclosures Related to Value

To further examine the usefulness of the GRI disclosures proposal with regard to accountability for the economic aspects collected in the reports, Table 4 indicates the 200 disclosures used by each university in the sample.

**Table 4:** Use of 200 standards in the sample universities.

University	200 Disclosures used	Maximum possible	Percentage of use
Università di Firenze	9	17	52.94%
Università degli Studi di Torino	6	17	35.29%
Fundación ESADE (Universidad Ramón Llull)	2	17	11.76%
Universidad de Cantabria	5	17	29.41%
Universidad de Cádiz	11	17	64.71%
Universidad Politécnica de Madrid (ETSII-UPM)	1	17	5.88%
ETH Zürich	1	17	5.88%
Universität Zürich	0	17	0.00%
IUNR Institut für Umwelt und Natürliche Ressourcen (IUNR-ZHAW)	2	17	11.76%
Manchester Metropolitan University	1	17	5.88%
TOTAL	38	170	22.35%

*Source: authors´ own elaboration*.

Table 4 shows a very different reflection of economic issues depending on the case study. While 6 of the 10 universities make residual use of these disclosures (reporting on between 0 and 2 of them), the other four show much greater use, reaching nine in the case of Firenze and up to 11 for Cádiz, out of a possible maximum of 17. It can be seen that these four universities are precisely those that provide more detail on their accountability in generated and distributed value (see Table 2 in the previous section).

Among the economic disclosures used, the most common is 201–1 (Direct economic value generated and distributed), reported by seven universities, usually referring to their annual accounts. This is followed by 201–4 (6 cases), where the public aid received is reported. Disclosure 201–1 is especially interesting as it refers to the generation of value, even if it is from an economic perspective. In this sense, significant differences in approach are observed between the seven universities that use it. In three of the cases (ESADE, ETSII-UPM and IUNR-ZHAW), the information replicates the usual information on the income statement (income and expenses), from a financial approach. In the case of Cádiz, an analysis of proximity purchases and payroll is added to the financial approach which, in part, reflects the economic value provided in these cases. However, in up to three cases (Cantabria, Firenze and Torino), the perspective is of generated and distributed value, which allows an approximation of the value generated for different stakeholders, an aspect which is given particular attention by Firenze and Torino. This shows that certain universities find the use of GRI disclosures to be a good practice for going beyond the presentation of their financial statements. This could serve to inspire other similar institutions.

## Discussion

Firstly, with regard to the identification of stakeholders in the reports, the preponderance of market and business-related institutions among the external stakeholders is remarkable. This result is in line with that found in the literature review, which showed that universities have a stronger relationship with those interest groups related to the economic system than with other interest groups more related to the social system. This trend seems to reflect a central focus of the universities in securing potential job opportunities for students. With regard to Government or Sector regulatory bodies, present in 90% of cases, their relevance may come both from the fact that they provide funds for research and teaching (90% of universities in the sample are public) and from the regulation that affects the entire sector.

Although not apparent in all the reports studied, there is some evidence that other agents more related to the social context, such as the local community and NGOs, and to the academic sector, such as investors and collaborators, are gradually being incorporated into these reports. However, and in line with findings from previous similar studies (Bellucci et al., 2019), these stakeholders are usually presented in a somewhat generic way. This confirms the general interest in improving collaboration with those actors with whom there is no specific functional relationship but, at the same time, this generality makes the interpretation of this relationship difficult. Examples include mention of the physical and social environment by the University of Cantabria, the territory mentioned by the Università di Firenze, and society by the University of Cádiz.

In terms of stakeholder relations channels, reference is often made to consultation processes, which, given their characteristics, do not really constitute two-way relationships (Pantoja et al., 2015). The lack of formality in these types of links makes them dependent on the context, with the characteristics of each organisation determining their composition and relevance. Moreover, only 30% of the cases show more stable channels, which make it easier for stakeholders to influence university activities along the lines of Mitchell et al. (1997). This could be a significant weakness for the university system since, as the literature has identified (Saurbier, 2021), it is a stable partnership which allows the incorporation of improvements in governance and decision-making processes at the institutional level.

In general, the GRI format seems appropriate for introducing the question of stakeholders and the necessary relationship with them for setting objectives (material topics) as well as a way of universities moving towards integrated reporting models.

However, and despite the fact that from its base (101, Foundations), it refers to the need to respond to the expectations and interests of the different stakeholders, there is no disclosure directly related to the measurement of social value, which may be why this issue has not been seen in the reports analysed in this study.

Nevertheless, the GRI methodology does promote the reporting of information on economic value generated, with disclosures, such as 201–1 (Direct economic value generated and distributed) or 203–2 (Significant indirect economic impacts), allowing for an extension of this concept, with a consensus on common methodologies, instruments or proxies so that the information provided by the universities is comparable.

In practice, no report in the sample refers to the ‘generated social value’ as such. The value generated by the universities is never expressed in monetary terms, as proposed by some authors (Retolaza et al., 2015; Ayuso et al., 2017). However, throughout all the reports, and in different GRI disclosures, information is provided on elements, such as employability, contracts and student body, that in certain studies already cited have been considered ‘value generating variables’.

Regarding the creation of value for stakeholders, the sample shows that some universities, such as Firenze or Torino, do include a calculation of these issues based on their financial statements. This could serve to promote these practices among other HEIs through benchmarking, and the use of this potential in a generalised way by universities.

It should also be noted that practically all the reports analysed cover aspects beyond what is envisaged by GRI, such as the approach taken in the institution towards the Sustainable Development Goals (SDG). Considering the few referrals made to the social value generated, the effort made in the reports to reflect the contribution of universities to the global challenges posed by the 2030 Agenda is quite encouraging.

## Conclusion

The evolution of the demand for greater transparency and improvements in the disclosure of non-financial information calls for new practices and new communication channels that allow organisations to express the value generated for their stakeholders.

While the academic literature reinforces the idea of universities committed to the generation of social value, as well as the need for its dissemination through integrated reports, and the importance of interaction with stakeholders (as a contrast process to identify the value generated), the results show that various issues are underrepresented in the GRI reports analysed. This is surprising, considering that the universities analysed here are likely leaders in their commitment to using the GRI as a wider reporting model.

However, it is clear that the objective of interacting with stakeholders, which is central to the preparation of GRI Reports, focuses on identifying aspects on which these wish to be informed rather than on identifying variables of value. On a positive note, it seems that once agile channels of communication with stakeholders have been established, these can be used to identify how value is generated and what aspects could be improved. The case studies analysed in this study, however, show that those communication channels which are unidirectional prevail and that the bidirectional ones have a high contingency component, which makes it difficult to introduce improvements and innovations in university management.

It is worth remembering, both for universities and academics who generate knowledge from their work, that this type of partnership is especially relevant in the current context of addressing the global challenges posed by society. All the strategic documents and global regulatory frameworks reflect this, such as the 2030 Agenda, which dedicates its SDG 17 to the necessary promotion of this type of partnership. To what extent the university can adapt its structures to a more organic relationship with its stakeholders and transfer the value that its activity generates to society are questions which require further empirical evidence. Identifying good practices and benchmarks among the universities that are already adapting integrated reporting models would be a step in the right direction.

Lastly, the results of the study should be interpreted taking into account certain limitations. On the one hand, the caveats inherent in every comparative case study mean that, by studying a larger number of cases rather than focusing on a single case study, in-depth knowledge is sacrificed. On the other hand, the possible biases derived from selection of the sample means that it is advisable not to generalise the results to all sectors of the university system, not even those in a European context. In fact, it is possible that certain elements, such as the location of the universities or their size, could influence the depth of information presented on the value generated for their stakeholders. Finally, the very format of the reports is another limitation, since most of the information is spread out across the report, and given the nature of this study, it has been preferable to review in greater detail the information provided in the corresponding GRI disclosures.

Some of these limitations could well be addressed through future lines of research, for example: by conducting case studies, perhaps on the universities that in this study have shown themselves to be leaders in providing more comprehensive information; or by combining these detailed studies with studies of a larger sample, in particular sectors or countries, such as Italy, given that the study seems to point to possible good practices in Italian universities; or conducting studies with content analysis methodology which can capture in a more thorough and quantitative way certain key communication elements present in the reports. All these approaches could contribute to the ongoing generation of empirical evidence on the practice of integrated reporting in universities and identify good practices in other organisations and regions.

## Data Availability Statement

Publicly available datasets were analysed in this study. This data can be found at: https://www.globalreporting.org/reportregistration/verifiedreports.

## Author Contributions

All authors listed have made a substantial, direct and intellectual contribution to the work, and approved it for publication.

## Funding

This research was funded by University of the Basque Country (UPV/EHU) US20/11.

## Conflict of Interest

The authors declare that the research was conducted in the absence of any commercial or financial relationships that could be construed as a potential conflict of interest.

## Publisher’s Note

All claims expressed in this article are solely those of the authors and do not necessarily represent those of their affiliated organizations, or those of the publisher, the editors and the reviewers. Any product that may be evaluated in this article, or claim that may be made by its manufacturer, is not guaranteed or endorsed by the publisher.
